# Supplementary material for: Environmental sensing by mature B cells is controlled by the transcription factors PU.1 and SpiB
Source: Nat Commun. 2017 Nov 10;8:1426. doi: 10.1038/s41467-017-01605-1 (PMC5681560; doi:10.1038/s41467-017-01605-1)
Supplement: Supplementary file 1 — Supplementary information [file 41467_2017_1605_MOESM1_ESM.docx]

**Supplementary Figure 1. SpiB regulates CD23 expression on follicular B cells.** (**A**) Representative flow cytometric analysis of splenocytes from SpiB KO (*Spi1*^fl/fl^ *Cd23*^+/+^ *Spib*^-/-^; black) and control (*Cd23*^T/+^; solid grey) mice showing the expression of CD23 on follicular B cells (B220^+^ IgD^+^ IgM^int^). Numbers indicate the mean fluorescence intensity (MFI) of CD23 for the example depicted. The full gating strategy is shown in Supplementary Fig. 8A. (**B**) Graph shows the MFI ± s.d. of CD23. Each circle represents the results from an individual mouse. P values compare the indicated samples (two tailed t test). **** P<0.0001.

**Supplementary Figure 2**. **Loss of PU.1 and SpiB leads to altered serum immunoglobulin titres.** Serum immunoglobulin concentrations of the indicated isotypes are plotted as the mean ± s.d. for each of the four genotypes examined. Each circle represents the measurement from an individual mouse. P values compare the indicated samples (two tailed t test). * P<0.05, ** P<0.01, **** P<0.0001.

**Supplementary Figure 3. PU.1 and SpiB function in follicular B cells.** Lymph node follicular B cells from mice of the indicated genotype were purified using the strategy described in Supplementary Fig. 9C. RNA was extracted and whole transcriptome sequencing performed on duplicates of each cell population. (**A**) The read coverage of representative tracks is provided, showing deletion of exon 5 of *Spi1* (PU.1, boxed) and exon 6 of *Spib* in the appropriate genotypes. The exon-intron structure of *Spi1* and *Spib* is shown below. Note that *Spi1* exon 5 is partially retained in PU.1 SpiB DKO follicular B cells. (**B**) The number of differentially expressed (DE) genes (defined as log_2_ fold change >0.6 fold, log_2_ RPKM >0, P <0.15) identified for each genotype is shown relative to control B cells. Genes upregulated in the absence of the indicated transcription factor(s) are shown by bars above the line while genes downregulated are shown below. (**C)** The number of DE genes associated with the indicated haematopoietic cell lineage using Enrichr analysis is shown. (**D**) Venn diagram showing the overlap between DE genes that were downregulated in the absence of the indicated transcription factor. Boxed genes are those associated with the follicular B cell lineage.

**Supplementary Figure 4**. **PU.1 and SpiB are required for BAFF-R expression.** (**A**) RNAseq data showing the expression (in RPKM) of *Tnfrsf13c* (encoding the BAFF receptor) in resting naïve follicular B cells of the four genotypes examined. (**B**) Representative flow cytometric analysis of resting naïve follicular B cells of the indicated genotype (black lines) relative to control B cells (*Cd23*^T/+^; solid grey). Numbers indicate the mean fluorescence intensity (MFI) of BAFF-R for the example depicted. (**C**) BAFF-R expression is graphed as the MFI ± s.d. Each circle represents the results from an individual mouse. P values compare the indicated samples (two tailed t test). * P<0.05, ** P<0.01. The full gating strategies are shown in Supplementary Fig. 9B and C, respectively.

**Supplementary Figure 5**. **Minimal antibody secreting cell formation after 48h stimulation with CD40L+IL-4.** (**A**) Resting lymph node B cells from mice of the indicated genotype were cultured in the presence of CD40L+IL-4 for 48h before flow cytometric analysis. Numbers in the boxes are the proportion of CD138^+^ antibody secreting cells (<1%) or activated B cells (>90%) present in the cultures. Data are representative of two independent experiments. (**B**) Resting lymph node B cells from mice of the indicated genotype were labelled with CTV and cultured in the presence of CD40L+IL-4 for 48h before flow cytometric analysis. The indicated genotypes are shown with black lines relative to controls (solid grey). Data are representative of two independent experiments. The full gating strategies are shown in Supplementary Fig. 9C and D, respectively.

**Supplementary Figure 6. Analysis of the function of PU.1 and SpiB in B cells cultured in LPS and IL-4.** (**A**) Resting lymph node B cells from mice of the indicated genotype were labelled with CTV and cultured in the presence of LPS+IL-4 for 4d before flow cytometric analysis. Numbers in the boxes are the proportion of CD138^+^ antibody secreting cells **(**ASCs). Data are representative of 3 independent experiments. (**B**) Quantitation of the total cell number and the number of ASCs cultured as in (**A**) is shown as the mean ± s.d. (**C**) Resting lymph node B cells from mice of the indicated genotype were cultured in the presence of LPS+IL-4 for 48h. RNA was extracted and whole transcriptome sequencing performed on duplicates of each cell population. The read coverage showing deletion (boxed) of exon 5 of *Spi1* (PU.1) and exon 6 of *Spib* in the appropriate genotypes is shown mapped to the exon-intron structure. (**D**) The expression (in RPKM) of important regulators of mature B cell and ASC biology is shown for each of the four genotypes examined. * Indicates genes that are DE relative to controls (DE genes defined as log_2_ fold change >0.6 fold, log_2_ RPKM >0, P <0.15). Gene names with PU.1 peaks (P <10^-6^) are indicated in red. The full gating strategies are shown in Supplementary Fig. 9A and C, respectively.

**Supplementary Figure 7.** **PU.1 and SpiB are required for the maintenance of the expression of several BCR signalling components (A) and several immune sensors (B) in cells activated with LPS+IL-4**. Gene expression (from Supplementary Fig. S6) for the indicated genotype following stimulation with LPS+IL-4 for 48h is plotted as fold change (log_2_) relative to the expression in the control genotype. * Indicates genes that are DE relative to controls (DE genes defined as log_2_ fold change >0.6 fold, log_2_ RPKM >0, P <0.15). Gene names in (A-B) with PU.1 binding in ChIPseq analysis (P<10^-6^) are indicated in red.

**Supplementary Figure 8. Gating strategies for data in Figures 1, 2, 3 and Supplementary Figure 1.** (**A**) Gating strategy for Fig. 1A and Supplementary Figure 1 for the detection of follicular B cells (B220^+^ IgD^+^ IgM^int^), CD23 expression and MZ (B220^+^ IgM^+^ IgD^low^ CD21^+^) B cells. (**B**) Gating strategy for Fig. 2A for the detection of splenic plasma cells (B220^-^ CD138^+^ CD98^+^). (**C**) Gating strategy for the detection of isotype switched B cells (B220^+^ IgM^-^ IgD^-^ Gr-1^-^) that bind NP or (**D**) express Bcl6. In each case, the gating strategy for the control population (*Cd23*^T/+^) is shown.

**Supplementary Figure 9. Gating strategies for data in Figures 4, 7, and Supplementary Figures 3, 4, 5, 6**. (**A**) Gating strategy for Fig. 4A, 7E and Supplementary Fig. 6A for the *in vitro* differentiation of B cells into antibody secreting cells (CD138^+^) or IgG1^+^ B cells. Note, calibration beads in the second panel are gated (2.9%) and were used to calculate cell numbers. Gating strategy is shown for the control population shown in Fig. 4A. (**B**) Gating strategy for Fig. 7B, C and Supplementary Fig. 4B for the detection of BAFF-R or CD40. Gating strategy is shown for the control population in Fig. 7C. (**C**) Gating strategy for Supplementary Fig. 3A, 4A, 5A, 6C for the isolation of naïve or activated B cells for RNAseq. Sorted B cells were CD3^-^ B220^+^ CD138^-^. Gating strategy is shown for the control population in Supplementary Fig. 5A. (**D**) Gating strategy for Supplementary Fig. 5B for the detection of B cell proliferation. Data shown is for the control population. Calibration beads in the first panel are gated (19.9%) and were used to calculate cell numbers. Gating strategy is shown for the control population shown in Fig. 4A.
