# Supplementary material for: Environmental sensing by mature B cells is controlled by the transcription factors PU.1 and SpiB
Source: Nat Commun. 2017 Nov 10;8:1426. doi: 10.1038/s41467-017-01605-1 (PMC5681560; doi:10.1038/s41467-017-01605-1)
Supplement: Supplementary file 3 — Description of Additional Supplementary Files [file 41467_2017_1605_MOESM3_ESM.pdf]

Description of Additional Supplementary Files

File Name: Supplementary Data 1

Description: **Identity of PU.1 and SpiB dependent genes**. The identity of genes differentially expressed (DE) in B cells cultured for 48h in the presence of CD40L+IL-4 between control and PU.1 KO (tab label, PU.1), control and SpiB KO (tab label, SpiB) and control and PU.1 SpiB DKO (tab label, PU.1 SpiB) genotypes, respectively. Shown for each DE gene is the $\log_2$ fold change between the genotypes, the adjusted P value (adj.P.Val) and the average $\log_2$ expression for all samples (AveExpr). DE genes were identified as $\log_2$fold change>0.6, RPKM (reads per kilobase of exon model per million mapped reads) >$\log_2$0 and P<0.15. Data are the mean of 2 biological replicates.
